# Oxaliplatin Depolarizes the IB4^–^ Dorsal Root Ganglion Neurons to Drive the Development of Neuropathic Pain Through TRPM8 in Mice

**DOI:** 10.3389/fnmol.2021.690858

**Published:** 2021-06-04

**Authors:** Bin Wu, Xiaolin Su, Wentong Zhang, Yi-Hong Zhang, Xinghua Feng, Yong-Hua Ji, Zhi-Yong Tan

**Affiliations:** ^1^Institute of Special Environment Medicine, Nantong University, Nantong, China; ^2^Department of Pharmacology and Toxicology, Stark Neurosciences Research Institute, Indiana University School of Medicine, Indianapolis, IN, United States; ^3^Department of Biochemistry and Molecular Biology, Indiana University School of Medicine, Indianapolis, IN, United States; ^4^Collaborative Innovation Center of Yangtze River Delta Region Green Pharmaceuticals, College of Pharmaceutical Sciences, Zhejiang University of Technology, Hangzhou, China; ^5^Laboratory of Neuropharmacology and Neurotoxicology, Shanghai University, Shanghai, China

**Keywords:** oxaliplatin, neuropathic pain, dorsal root ganglion, IB4, initial drive, membrane depolarization, TRPM8

## Abstract

Use of chemotherapy drug oxaliplatin is associated with painful peripheral neuropathy that is exacerbated by cold. Remodeling of ion channels including TRP channels in dorsal root ganglion (DRG) neurons contribute to the sensory hypersensitivity following oxaliplatin treatment in animal models. However, it has not been studied if TRP channels and membrane depolarization of DRG neurons serve as the initial ionic/membrane drives (such as within an hour) that contribute to the development of oxaliplatin-induced neuropathic pain. In the current study, we studied in mice (1) *in vitro* acute effects of oxaliplatin on the membrane excitability of IB4^+^ and IB4^–^ subpopulations of DRG neurons using a perforated patch clamping, (2) the preventative effects of a membrane-hyperpolarizing drug retigabine on oxaliplatin-induced sensory hypersensitivity, and (3) the preventative effects of TRP channel antagonists on the oxaliplatin-induced membrane hyperexcitability and sensory hypersensitivity. We found (1) IB4^+^ and IB4^–^ subpopulations of small DRG neurons displayed previously undiscovered, substantially different membrane excitability, (2) oxaliplatin selectively depolarized IB4^–^ DRG neurons, (3) pretreatment of retigabine largely prevented oxaliplatin-induced sensory hypersensitivity, (4) antagonists of TRPA1 and TRPM8 channels prevented oxaliplatin-induced membrane depolarization, and (5) the antagonist of TRPM8 largely prevented oxaliplatin-induced sensory hypersensitivity. These results suggest that oxaliplatin depolarizes IB4^–^ neurons through TRPM8 channels to drive the development of neuropathic pain and targeting the initial drives of TRPM8 and/or membrane depolarization may prevent oxaliplatin-induce neuropathic pain.

## Introduction

The third-generation platinum drug oxaliplatin is the most common used drug for locally advanced and metastatic cancer of the colon, rectum, or pancreas. Compared to classic platinum agents, such as cisplatin, oxaliplatin has lower hematotoxicity and gastrointestinal toxicity (de Gramont et al., 2000). However, unlike other platinum agents which cause the chronic neuropathy, oxaliplatin specifically induced acute painful chemotherapy-induced peripheral neuropathy (CIPN) during or within hours after its first infusion in almost 90% patients (Leonard et al., 2005; Gebremedhn et al., 2018). The acute pain may become increasingly severe in the subsequent cycles and can become chronic that lasts for months and years (Beijers et al., 2014; Briani et al., 2014). The oxaliplatin-induced CIPN is evoked or exacerbated by cold, with symptoms including throat discomfort, paresthesia and dysesthesia of the hands, feet, and perioral region; these symptoms often lead to reduction or discontinued use of oxaliplatin treatment (Argyriou et al., 2010; Kerckhove et al., 2017).

The mechanisms of oxaliplatin-induced CIPN include DNA damage, mitochondrial dysfunction, calcium chelation, reactive oxygen species (ROS) production, and ion channel remodeling in dorsal root ganglion (DRG) neurons (Joseph et al., 2008; Descoeur et al., 2011; Boyette-Davis et al., 2015; Viatchenko-Karpinski et al., 2018; Rovini, 2019; Trecarichi and Flatters, 2019). For instance, transient receptor potential (TRP) channels expressed on DRG neurons such as TRPV1, TRPA1, and TRPM8 have been studied following the treatment of oxaliplatin (Chukyo et al., 2018). Several studies have shown that the mRNA expression level of TRPA1 and TRPM8, but not TRPV1 were upregulated within several days after oxaliplatin treatment (Gauchan et al., 2009; Ta et al., 2010; Yamamoto et al., 2015; Nakagawa and Kaneko, 2017). More importantly, mechanical allodynia and cold hypersensitivity induced by single or repeated administration of oxaliplatin was abolished by pharmacological inhibition or a gene deficiency of TRPA1 or TRPM8 (Gauchan et al., 2009; Nassini et al., 2011). These findings suggest that TRPA1 and/or TRPM8 channels play important role in the maintaining of oxaliplatin-induced neuropathic pain several days following oxaliplatin treatment. On the other hand, it has not been studied if TRP channels and membrane depolarization of DRG neurons serve as the initial ionic/membrane drives (such as within an hour) that contribute to the development of oxaliplatin-induced neuropathic pain. In the current study, we investigated the role of TRP channels and membrane depolarization in the initial pain-driving process following oxaliplatin treatment using *in vitro* and *in vivo* mouse models. We characterized the membrane excitability of IB4^+^ and IB4^–^ subpopulations of small DRG neurons, examined the responses of IB4^+^ and IB4^–^ DRG neurons to acute oxaliplatin, and studied the effects of antagonists of membrane depolarization and subtypes of TRP channels on the membrane hyperexcitability and/or pain behaviors induced by oxaliplatin. We found that IB4^+^ and IB4^–^ subpopulations of small DRG neurons displayed large differences in membrane excitability and that oxaliplatin selectively depolarized IB4^–^ neurons. Antagonists of TRPA1 or TRPM8 prevented oxaliplatin-induced membrane depolarization and that targeting membrane depolarization or TRPM8 prevented oxaliplatin-induced neuropathic pain behaviors. There results suggest that TRPM8 and membrane depolarization in IB4^–^ neurons might serve as the initial ionic/membrane drives that contribute to the development of oxaliplatin-induced neuropathic pain.

## Materials and Methods

### Animals

Male C57BL/6 mice at the age of 8 weeks (Jackson Laboratory, Bar Harbor, ME, United States) were used for cell culture and behavior experiments. Mice were housed five or less per cage at a temperature-controlled room (22 ± 0.5°C, 12 h/12 h light/dark cycle) and were with free access to water and pellet diet. All experimental protocols were approved by the Institutional Animal Care and Use Committees of the Indiana University School of Medicine, Indianapolis, IN, United States. All procedures were conducted in accordance with the Guide for Care and Use of Laboratory Animals published by the National Institutes of Health and the ethical guidelines established by the International Association for the Study of Pain.

### Drugs and Their Administration

Oxaliplatin and Capsazepine were purchased from Sigma-Aldrich (St. Louis, MO, United States). Retigabine was purchased from Alomone Labs (Jerusalem, Israel), A-967079 was purchased from MedChemExpress (Monmouth Junction, NJ, United States), and TC-I was purchased from Tocris, Bio-Techne Corporation (Minneapolis, MN, United States). For electrophysiology, stock solutions were made in distilled water (oxaliplatin, 10 mM) or DMSO (A-967079, capsazepine, and TC-I). The stock solutions were diluted by bath solution to reach the final concentration of chemicals including oxaliplatin (50 μM), A-967079 (1 μM), capsazepine (10 μM) and TC-1 (10 nM). For chemicals diluted from DMSO stocks, the final solution contains 0.1% DMSO. All these drugs were pre-treated in the bath solution for 15–60 min before electrophysiological recordings. For behavior test, stock solutions of oxaliplatin in distilled water (5 mg/ml), were further diluted in 5% glucose to give the final concentration of 1.25 mg/ml. Retigabine, A-967079, TC-1 were dissolved in DMSO and further diluted in saline (to give 2% DMSO) for different final concentrations. A single intraperitoneal (i.p.) injection of oxaliplatin (5 mg/kg) was conducted and retigabine (10 mg/kg, i.p.), TC-I (10 mg/kg, i.p.) and A-967079 (100 mg/kg, p.o.) were administrated daily for 5 days from 2 days before to 2 days after the injection of oxaliplatin. Chemicals without specified sources were from Sigma-Aldrich (St. Louis, MO, United States).

### Behavior Test

All the testing was carried out in accordance with the approved guidelines. All behavioral measurements were performed in conscious, unrestrained, and age-matched adult male mice.

#### Mechanical Nociceptor Assay

As described previously, the von Frey assay of “simplified up-down” method was used to assess mechanical sensitivity (Su et al., 2019). Mice were placed in Plexiglas cubicle containers on a metal mesh wire platform to allow access to the plantar hindpaw. A set of eight calibrated von Frey filaments ranging from 0.008 to 6 g (North Coast Medical, Morgan Hill, CA, United States) were applied alternately to the plantar surface of each hindpaw until they bent. The duration of each stimulus was approximately 1 s and nociceptive behaviors included retraction/lifting, rapid shaking, and/or licking of the hindpaw.

#### Heat Nociceptor Assay

The thermal sensitivity was assessed by applying infrared heating to the plantar surface of hindpaw and the response latency was read from an automated device (IITC model 400, Woodland Hills, CA, United States), as described previously (Zhou et al., 2019). Each hindpaw was tested five times with 5 min interval, and the withdrawal latency was averaged. To avoid tissue damage by prolonged thermal stimuli, cut off latency was set as 20 s.

#### Cold Nociceptor Assay

The acetone test was performed as previously described (Su et al., 2019). Mice were placed to the same setting described above for the von Frey test. Fifty microliter of acetone was applied to the center of the ventral side of the hindpaw and responses were observed. In the first 20 s following acetone application, if the mouse did not withdraw, flick or stamp of the paw then 0 points were recorded for the trial. However, if within this 20 s period the animal responded to acetone, then the animal’s response was assessed for an additional 20 s. Responses to acetone were graded according to the following 4-point scale: 0, no response; 1, quick withdrawal, flick or stamp of the paw; 2, prolonged withdrawal or repeated flicking of the paw; 3, repeated flicking of the paw with licking directed at the paw. Acetone test was applied alternately three times to each paw and the responses scored categorically.

#### Rotarod Test

The rotarod test was conducted according to the previous publication (Liu et al., 2014). The animals had two sessions of training tests before the first scheduled test. At each training or scheduled test, three levels of rotating speed were used: 1–18 rpm, 3–30 rpm, and 4–40 rpm. The time for the animal to stay on the rod from the beginning of rod-rotation to the falling of the animal to the ground was recorded. A cut-off time of 120 s was used.

### Cell Culture

Dorsal root ganglion neurons were dissociated and prepared from adult mice using a similar protocol as previously described (Su et al., 2019). Briefly, mice were sacrificed by exposure to CO_2_ and decapitated. DRG were rapidly removed and placed in Puck’s solution containing digesting enzymes. The DRGs were digested with Liberase TM (0.35 U/ml; Sigma-Aldrich, St. Louis, MO, United States) for 35–40 min before another 10 min with Liberase TL (0.25 U/ml; Sigma-Aldrich, St. Louis, MO, United States) and papain (30 U/ml, Worthington Biochemical) at 37°C. The ganglia were then triturated with fire-polished Pasteur pipettes. The dispersed cells were resuspended in F12 (Thermo Fisher Scientific, Waltham, MA, United States) medium supplemented with 10% FBS (Thermo Fisher Scientific, Waltham, MA, United States) and 1% penicillin/streptomycin (MediaTech, Inc., Manassas, VA, United States) and plated on coverslips coated with polyornithine (Neuvitro Corporation, Vancouver, WA, United States) and laminin. Cell cultures were maintained in regular 95% air and 5% CO_2_ at 37°C in an incubator.

### Electrophysiological Recordings

Dorsal root ganglion neurons were recording 16–24 h after dissociation as described previously (Su et al., 2019; Wu et al., 2019). Small diameter DRG neurons (<25 μm) were chosen for whole-cell patch clamp recording in the current-clamp mode at room temperature. A perforated whole-cell patch clamp recording was conducted by including 240 μg/mL Amphotericin B in the pipette solution (Tan et al., 2010). The bath solution consisted of 140 mM NaCl, 3 mM KCl, 2 mM MgCl2, 2 mM CaCl2, 10 mM HEPES, pH 7.3. DRG neurons were recorded with fire-polished, borosilicate glass patch pipettes (5–8 MΩ), which were pulled from borosilicate glass capillaries (Harvard Apparatus, Holliston, MA, United States) using a Sutter P-97 puller (Sutter Instrument, Novato, CA, United States). The pipette solution contained 30 mM KCl, 110 mM potassium gluconate, 0.5 mM EGTA, 5 mM HEPES, and 3 mM Mg-ATP, pH 7.3. Data were acquired using Axopatch 200B patch-clamp amplifier (Molecular Devices Corporation, Sunnyvale, CA, United States) driven by a personal computer in conjunction with an A/D and D/A board (DigiData 1320 A series interface, Molecular Devices Corporation). The action potential (AP) was evoked by depolarizing current steps with long or short time durations (1,000 ms or 5 ms). The long current steps were used to test AP rheobases and AP numbers. The short current steps were used to trigger single action potential that was used to calculate the shape properties of APs. Signals were low-pass filtered at 5 kHz, sampled at 20 kHz and analyzed offline. To avoid the confounding effects of IB4 staining on the forming of Giga-seal for patch clamp recording and potentially on the electrophysiological properties of DRG neuron, DRG neurons were stained by IB4-FITC (5 μg/ml, incubated for 10 min) immediately after recording to distinguish IB4^+^ and IB4^–^ neurons (Dirajlal et al., 2003; Vilceanu et al., 2010).

### Quantification and Statistics

GraphPad Prism v5.0 was used for statistical analyses. All the results were presented as mean ± SEM. The behavioral data were analyzed by two-way repeated measures (RM) ANOVA followed by Bonferroni test. The electrophysical recording data were analyzed by Student’s *t*-test or two-way RM ANOVA followed by Bonferroni test. For all experiments, *P* < 0.05 was considered to be significant (^∗^*P* < 0.05, ^∗∗^*P* < 0.01, and ^∗∗∗^*P* < 0.001).

## Results

### IB4^+^ and IB4^–^ Neurons Showed Substantially Different Electrophysiological Properties

**FIGURE 1:**
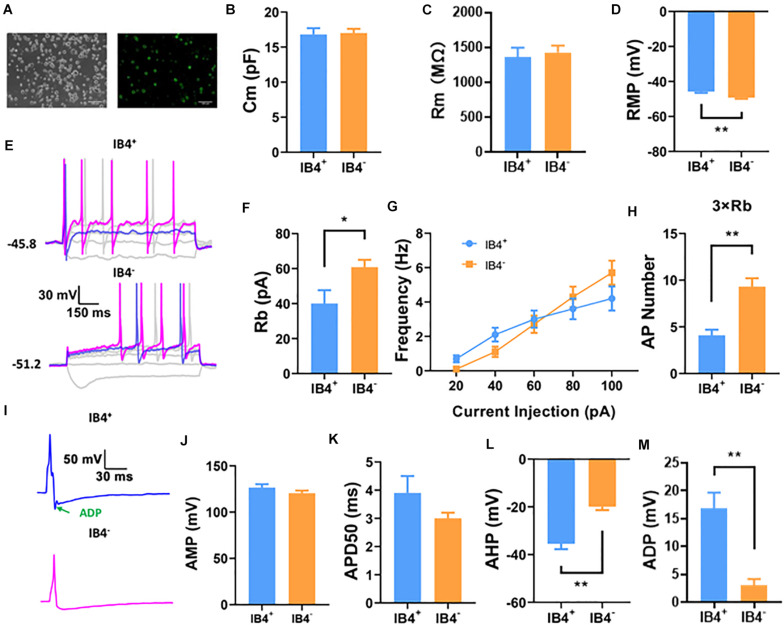
Comparison of passive and active properties of IB4^+^ and IB4^–^ subpopulations of small DRG neurons in mouse. IB4^+^ and IB4^–^ subpopulations of small DRG neurons (<25 μm), separated by the staining of IB4-FITC **(A)**. Small DRG neurons were recorded in current-clamp mode using a perforated configuration of whole-cell patch clamping. To avoid the confounding effects of IB4 staining on the forming of Giga-seal for patch clamp recording and potentially on the electrophysiological properties of DRG neurons, the DRG neurons were stained immediately after recording. Representative recordings using a long (1,000 ms) or short (5 ms) step current protocol were shown in **(E,I)**, respectively. Passive properties including membrane capacitance [Cm, **(B)**], membrane resistance [Rm, **(C)**], resting membrane potential [RMP,**(D)**], and active properties including rheobase [Rb, **(F)**], frequency **(G)**, number at 3x rheobase **(H)**, amplitude [AMP, **(J)**], half duration [APD50, **(K)**], afterhyperpolarization [AHP, **(L)**], and afterdepolarization [ADP, **(I,M)**] of action potentials were compared between IB4^+^ and IB4^–^ neurons. *n* = 17–38 for each groups (see detailed numbers for each group in results). *, *P* < 0.05; **, *P* < 0.01; Student’s *t*-test.

IB4^+^ and IB4^–^ neurons are two major cell types of small diameter (<25 μm) DRG neurons, most of which are nociceptive. To begin with the study, we compared the electrophysiological properties of small IB4^+^ (*n* = 21) and IB4^–^ (*n* = 38) DRG neurons using a perforated current-clamp whole-cell recording (Figure 1A). As shown in Figure 1, the membrane capacitance (Cm, Figure 1B) and membrane resistance (Rm, Figure 1C) were similar between IB4^+^ and IB4^–^ neurons. Compared to IB4^–^ neurons, the resting membrane potentials in IB4^+^ neurons were significantly depolarized and the rheobase currents were lower (Figures 1D–F). Although the number of action potentials, triggered by current injections from 20 to 100 pA were not significantly different (Figure 1G), a suprathreshold current injection (at 3 × rheobase) elicited significantly more action potentials in IB4^–^ neurons than those in IB4^+^ ones (Figure 1H).

We further compared the properties of single action potentials elicited in IB4^+^ (*n* = 17) and IB4^–^ (*n* = 24) neurons (Figure 1I). Although the amplitude (Figure 1J), duration (Figure 1K), the maximal rising slope (68.8 ± 4.3 mV/ms in IB4^+^ cells and 70.9 ± 4.0 mV/ms in IB4^–^ cells) or decaying slope of action potentials (−55.7 ± 3.3 mV/ms in IB4^+^ cells and −64.0 ± 2.9 mV/ms in IB4^–^ cells) were not significantly different between these two cell subtypes, the afterhyperpolarization of action potentials in IB4^+^ was nearly doubled compared to that in IB4^–^ neurons (Figure 1L). Moreover, the action potential in IB4^+^ neurons exhibited an apparent afterdepolarization potential (ADP) spike, which is often absent in IB4^–^ neurons (Figures 1I,M). Taken together, our results suggest that the electrophysiological properties between mouse IB4^+^ and IB4^–^ neurons are substantially different.

### Oxaliplatin Selectively Depolarized IB4^–^ Neurons, but Not IB4^+^ Neurons

**FIGURE 2:**
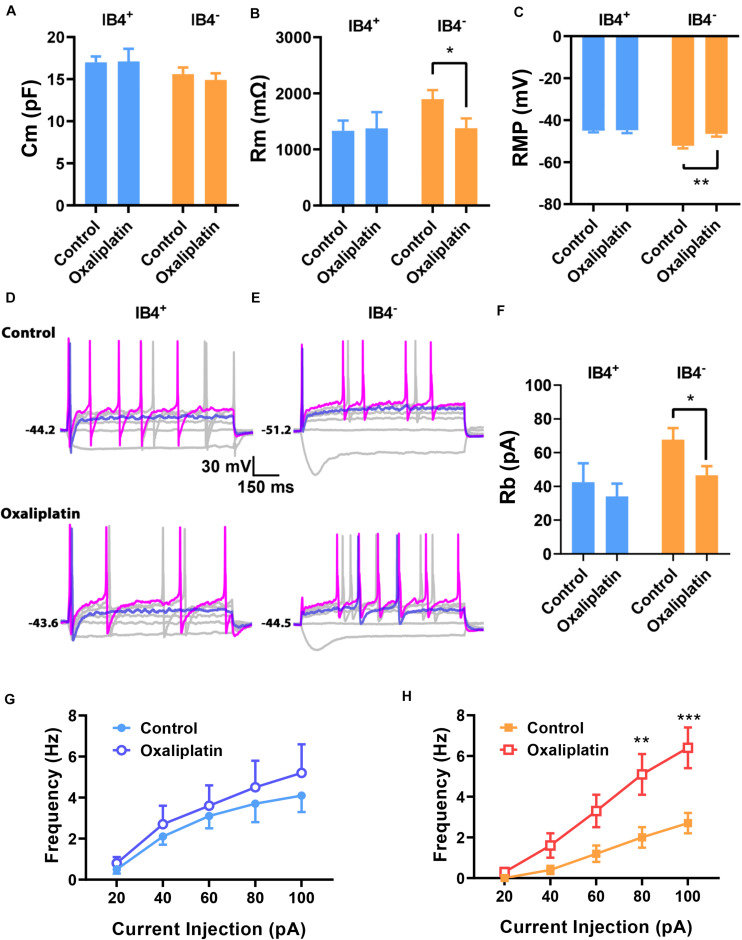
Effects of oxaliplatin on the electrophysiological properties of IB4^+^ and IB4^–^ subpopulations of small DRG neurons. Oxaliplatin (50 μM) was pre-treated in the recording chamber for 15–60 min. The passive and active properties of IB4^+^ and IB4^–^ subpopulations of small DRG neurons were recorded and presented in the same way described in the legend of Figure 1. Oxaliplatin did not change the Cm of both IB4^+^ and IB4^–^ neurons **(A)** but decreased the Rm **(B)** and depolarized the RMP **(C)** in IB4^–^ neurons; **(D,E)** Representative recordings from IB4^+^ or IB4^–^ DRG neurons in the absence or presence of oxaliplatin, respectively. **(F)** The effect of oxaliplatin on rheobase of IB4^+^ and IB4^–^ neurons. **(G,H)** The effect of oxaliplatin on the firing frequency of IB4^+^ (G) and IB4^–^ (H) neurons, respectively. *n* = 8–14 for each group (see detailed numbers for each group in results). *P < 0.05, **P < 0.01, ***P < 0.001, Student’s *t*-test **(B,C,F)** or Two-way repeated measure ANOVA **(H)**.

To assess the effects of oxaliplatin on the electrophysiological properties in the two subgroups of small DRG neurons, IB4^+^ and IB4^–^ DRG neurons were recorded after 15–60 min pre-treatment of oxaliplatin (50 μM) *in vitro*. The cells in control (*n* = 12 and 13 for IB4^+^ and IB4^–^ neurons, respectively) and oxaliplatin (*n* = 8 and 14 for IB4^+^ and IB4^–^ neurons, respectively) groups have similar membrane capacitance for both IB4^+^ and IB4^–^ groups (Figure 2A). In addition, oxaliplatin did not change the membrane resistance (Figure 2B), resting membrane potential (Figure 2C), the rheobase and the firing frequency of action potentials in IB4^+^ neurons significantly (Figures 2D,F,G). In contrast, the membrane resistances of IB4^–^ neurons deceased by ∼500 MΩ after oxaliplatin treatment (Figure 2B). Meanwhile, the resting potentials of IB4^–^ neurons were depolarized by ∼5.6 mV following oxaliplatin treatment (Figure 2C), which consequently reduced the rheobase current and increased the firing frequency of action potentials trigged by 60–100 pA of injection currents (Figures 2E,F,H). The other parameters of action potentials such as AP threshold, amplitude, half-width, and afterhyperpolarization were not significantly changed by oxaliplatin in both cell subtypes (Table 1). Overall, these results indicate that the excitability of IB4^–^ neurons, but not IB4^+^ neurons, were selectively enhanced by acute oxaliplatin treatment *in vitro*.

**TABLE 1:** Shape properties of action potentials in the absence and presence of oxaliplatin.

IB4^+^	Vt (mV)	AMP (mV)	AHP (mV)	APD50 (ms)
Control (*n* = 12)	−13.8 ± 4.2	125.9 ± 4.6	−37.8 ± 2.8	3.4 ± 0.2
Oxaliplatin (*n* = 8)	−18.1 ± 3.0	128.7 ± 6.5	−30.5 ± 1.4	3.6 ± 0.6
**IB4^–^**				
Control (*n* = 13)	−17.0 ± 2.5	118.4 ± 5.0	−21.9 ± 2.4	3.4 ± 0.3
Oxaliplatin (*n* = 14)	−12.2 ± 2.8	112.3 ± 2.3	−20.6 ± 2.3	3.5 ± 0.3

*Vt, voltage threshold; AMP, amplitude; AHP, afterhyperpolarization; APD50, half duration.*

### Retigabine Prevented Oxaliplatin-Induced Nociceptive Behavior

To test if the oxaliplatin-induced membrane depolarization of DRG neurons might serve as an initial drive that contributes to the development of oxaliplatin-induced sensory hypersensitivity, we examined the potential preventative effects of retigabine, an opener of potassium channel Kv7 that hyperpolarizes the resting potential of DRG neurons (Corbin-Leftwich et al., 2016), on the sensory and motor behaviors in oxaliplatin treated mice.

**FIGURE 3:**
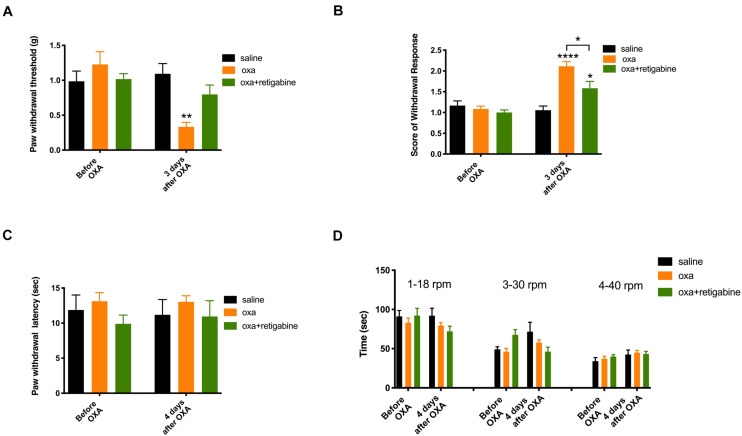
Effects of retigabine on the sensory and motor behaviors following oxaliplatin treatment. Oxaliplatin (oxa, 5 mg/kg, ip) was administrated at Day 0. Retigabine (10 mg/kg, ip, daily) was treated at Day –2, Day –1, Day 0, Day 1, and Day 2. The same groups of animals were tested for mechanical and cold behaviors at Day 3, and were tested for heat and motor behaviors at Day 4. **(A)** Paw withdrawal threshold to Von-Frey filament; **(B)** Score of withdrawal response to acetone; **(C)** Paw withdrawal latency to radiant heat; and **(D)** Time stayed on rod at different speeds in rotarod tests (*n* = 6 for all groups). *, *P* < 0.05; **, *P* < 0.01; ****, *P* < 0.0001; two-way repeated measure ANOVA.

As shown in Figure 3 (*n* = 6 for all groups), oxaliplatin significantly reduced the paw withdrawal threshold to mechanical stimuli (Figure 3A) and increased withdrawal score to cold stimuli (Figure 3B) 3 days after oxaliplatin treatment (5 mg/kg, ip). In contrast to the mechanical allodynia and cold hyperalgesia, oxaliplatin did not change the paw withdrawal latency to heat stimuli (Figure 3C) or time stayed on rod for the rotarod test (Figure 3D) 4 days following oxaliplatin, in the same groups of animals that had their mechanical and cold tests on Day 3 (Figures 3A,B). Although heat and motor behaviors were tested at Day 4 in these groups of animals in order to compare different types of behaviors in same groups of animals, separate experiments found that heat and motor behaviors were not changed by oxaliplatin at Day 3 as well (data not shown).

To test the potential preventative effects on oxaliplatin-induced sensory hypersensitivity, retigabine (10 mg/kg, ip, daily) was administrated at Day −2, Day −1, Day 0, Day 1, and Day 2. Oxaliplatin was injected at Day 0. As shown in Figure 3, retigabine largely prevented mechanical allodynia (Figure 3A) and significantly attenuated cold hyperalgesia (Figure 3B) caused by oxaliplatin. On the other hand, retigabine did not change heat or motor behaviors significantly (Figures 3C,D).

### TRP Channels Mediated the Depolarization Induced by Oxaliplatin

**FIGURE 4:**
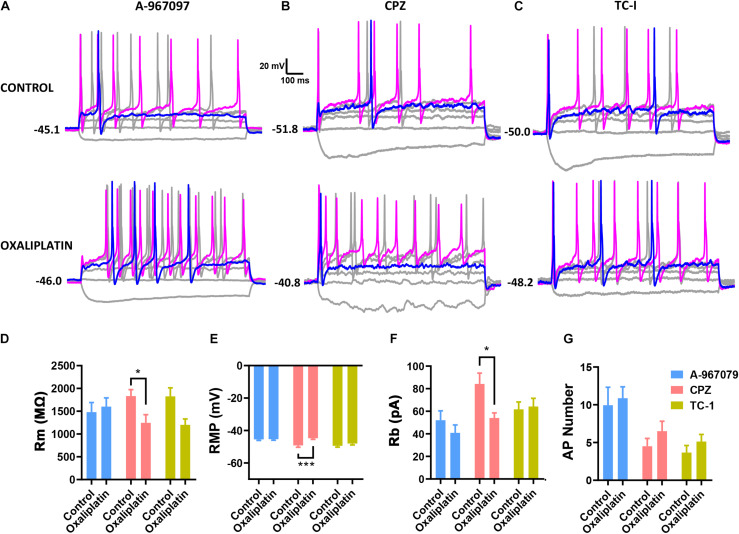
Effects of selective TRP channel antagonists on the electrophysiological properties of IB4^+^ and IB4^–^ subpopulations of small DRG neurons. Selective antagonists for TRPA1 (1 μM A-967097), TRPV1 (10 μM Capsazepine, CPZ), or TRPM8 (10 nM TC-I) were pre-treated in the recording chamber for 15 min before the addition of 50 μM oxaliplatin. The passive and active properties of IB4^+^ and IB4^–^ subpopulations of small DRG neurons were recorded and presented in the same way described in the legend of Figure 1. **(A-C)** Representative recordings in the absence or presence of oxaliplatin, and in the presence of A-967097, Capsazepine, and TC-I, respectively. Effect of oxaliplatin on Rm **(D)**, RMP **(E)** and Rb **(F)** in each group of TRP antagonists. **(G)** Number of action potentials evoked by 100 pA current injection in each group of cells. *n* = 6–12 for each group (see detailed numbers for each group in results). *P < 0.05, ***P < 0.001, Student’s *t*-test.

As the membrane depolarization may be the key electro- physiological effect caused by oxaliplatin that consequently results in the neuronal hyperexcitability (Figure 2), we further studied the possible ion channels that may be involved in the oxaliplatin-induced membrane depolarization. Because the membrane resistance of IB4^–^ neurons was significantly reduced by oxaliplatin along with the membrane depolarization, it is suggested that oxaliplatin increased the permeability of extracellular cations, such as sodium and calcium, at resting state. As TRP channels are a major family of non-selective cation channels expressed in DRG neurons that are directly involved in a variety of chemical and thermal sensing, we tested whether some of the major TRP channel subtypes are involved in the oxaliplatin-induced membrane depolarization. Specific channel antagonists including A-967079 (1 μM) (Figure 4A), Capsazepine (10 μM) (Figure 4B), and TC-I (10 nM) (Figure 4C), were used to block TRPA1, TRPV1, and TRPM8 channels, respectively.

As shown in Figure 4, pre-treatment of Capsazepine (*n* = 10 and 11 for control and oxaliplatin groups, respectively) did not prevent the oxaliplatin-induced changes in membrane resistance (Figure 4D), resting membrane potential (Figure 4E), AP rheobase (Figure 4F). However, in the presence of A-967079 (*n* = 12 and 10 for control and oxaliplatin groups, respectively) or TC-I (*n* = 6 and 12 for control and oxaliplatin groups, respectively), there were no significant changes in these parameters between control and oxaliplatin groups (Figures 4D–F). These results indicate that TRPA1 and/or TRPM8, but not TRPV1, may contribute to the oxaliplatin-induced membrane depolarization and neuronal sensitization, which might contribute to the initiation of sensory hypersensitivity induced by oxaliplatin.

### TC-I, but Not A-967079, Prevented Oxaliplatin-Induced Nociceptive Behavior

To test if TRPM8 and TRPA1 might be involved in the initial pain-driving process of the oxaliplatin-induced sensory hypersensitivity, we examined the potential preventative effects of TC-I and A-967079 on the sensory and motor behaviors in oxaliplatin treated mice.

**FIGURE 5:**
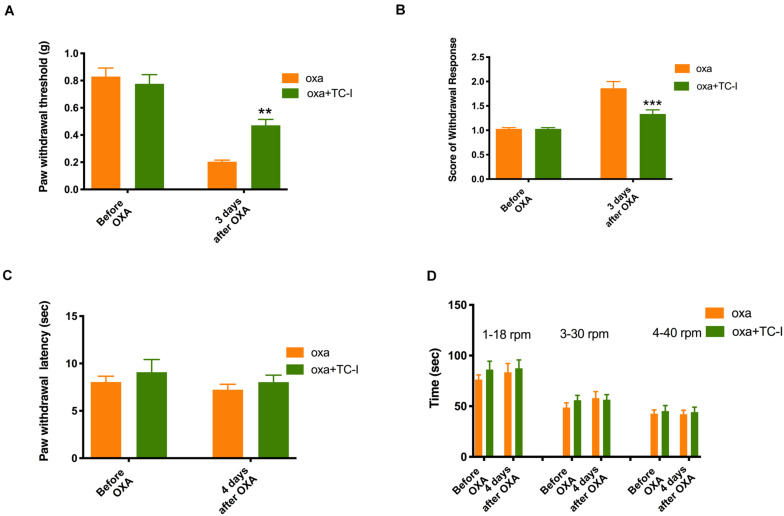
Effects of TC-I on the sensory and motor behaviors following oxaliplatin treatment. The administration of oxaliplatin, the schedule for TC-I (10 mg/kg, ip) treatment (same to the Retigabine treatment), the testing schedule for different types of behaviors were described in the Legend of Figure 3. **(A)** Paw withdrawal threshold to Von-Frey filament; **(B)** Score of withdrawal response to acetone; **(C)** Paw withdrawal latency to radiant heat; and **(D)** Time stayed on rod at different speeds in rotarod tests (*n* = 6 for all groups). **, *P* < 0.01; ****, *P* < 0.0001; two-way repeated measure ANOVA.

**FIGURE 6:**
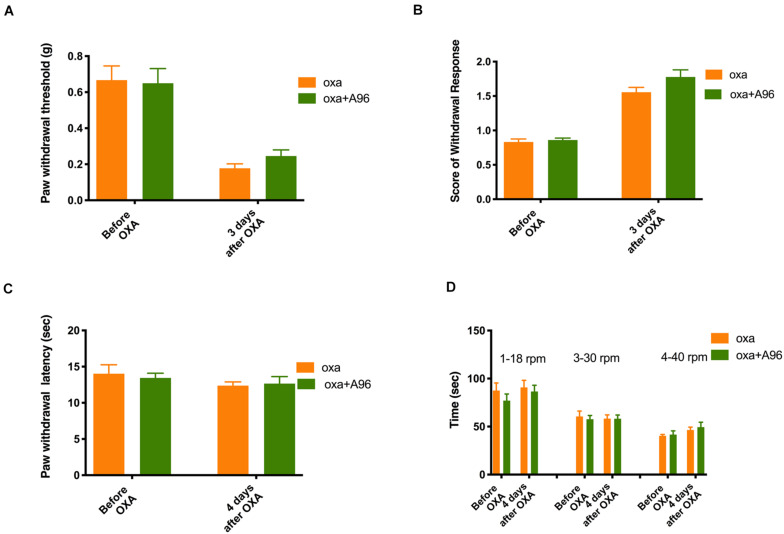
Effects of A-967079 on the sensory and motor behaviors following oxaliplatin treatment. The administration of oxaliplatin, the schedule for A-967079 (A-96, 100 mg/kg, po) treatment (same to the Retigabine and TC-I treatment), the testing schedule for different types of behaviors were described in the Legend of Figure 3. **(A)** Paw withdrawal threshold to Von-Frey filament; **(B)** Score of withdrawal response to acetone; **(C)** Paw withdrawal latency to radiant heat; and **(D)** Time stayed on rod at different speeds in rotarod tests (*n* = 6 for all groups).

As shown in Figure 5 (*n* = 6 for all groups), daily treatment of 10 mg/kg TC-I (ip, administrated at Day −2, Day −1, Day 0, Day 1, and Day 2) largely prevented mechanical and cold hypersensitivity induced by oxaliplatin administrated at Day 0 (Figures 5A,B) without causing any changes in the heat and motor behaviors tested (Figures 5C,D). In contrast, the treatment of 100 mg/kg A-967079 (po) with the same schedule of TC-I did not change any behaviors following oxaliplatin administration (Figure 6) (*n* = 6 for all groups). These results suggest that TRPM8 may play a critical role in the initiation of sensory hypersensitivity induced by oxaliplatin.

## Discussion

In the present study, we found that there were previously undiscovered, substantial differences in membrane excitability between IB4^+^ and IB4^–^ subpopulations of small-sized DRG neurons in mice. These differences included depolarized resting membrane potential, lower rheobase of the action potential, overwhelmingly larger afterdepolarization, and nearly doubled afterhyperpolarization in IB4^+^ neurons compared to IB4^–^ neurons (Figure 1). Differential properties of membrane excitability have been reported previously between IB4^+^ and IB4^–^ subpopulations of small DRG neurons of rats and mice (Stucky and Lewin, 1999; Wu and Pan, 2004; Choi et al., 2007; Zhang et al., 2010). These differences, in IB4^+^ compared to IB4^–^ subpopulation of neurons, included a longer duration of the action potential in both rat and mouse neurons, a hyperpolarized resting membrane potential and/or higher rheobases of action potentials in rat neurons, and a smaller afterhyperpolarization in cutaneous rat neurons. Interestingly, none of the major findings in the present study have been reported previously. A possible reason for the different findings between previous and current studies could be due to the different recording configuration of whole-cell patch clamping. Typical whole-cell recordings were used in the previous studies while a perforated whole-cell recording was used in the present study. As the perforation introduced by amphotericin B does not allow the exchange of ions and molecules larger than monovalent ions between patch pipettes and cytosols of cells, divalent ions such as intracellular Ca^2+^, and other cytosolic molecules might contribute to these differences. For example, a predominant expression of large conductance, calcium activated potassium currents (BK_Ca_) was discovered in IB4^+^ cutaneous DRG neurons which would result in a larger afterhyperpolarization in these neurons (Zhang et al., 2010). However, a smaller afterhyperpolarization was actually observed in these neurons using typical whole-cell recording (Zhang et al., 2010). On the other hand, using perforated whole-cell recording, we found that there were a nearly doubled afterhyperpolarization expressed in the IB4^+^ DRG neurons (Figures 1I,L). Therefore, the revealing of the larger afterhyperpolarization in IB4^+^ neurons is likely due to the perforated whole-cell recording used in the current study that allowed the endogenous Ca^2+^ dynamics unbuffered by the Ca^2+^ chelators included in the patch pipette. In addition to the different configurations of whole-cell recordings, other factors such as species of rodents, mouse strains, culture protocols could also contribute to the differences among previous and current studies.

Among the major differential membrane properties between IB4^+^ and IB4^–^ neurons found in the current study, the afterdepolarization recorded in the small DRG neurons has not been reported (Figures 1I,M). Previously a different type of afterdepolarization was reported in the subpopulations of medium, or small-to-medium DRG neurons that are enriched with T-type calcium channels (Ca_T_) in rats (White et al., 1989; Nelson et al., 2005). However, the afterdepolarization recorded from Ca_T_-enriched neurons did not appear at the resting membrane potential and a membrane hyperpolarization was needed to induce this type of afterdepolarization. On the other hand, afterdepolarization recorded in the small mouse neurons of the present study was induced at normal resting membrane potential. Moreover, the afterdepolarization was much shorter in the present study compared to that recorded in the previous studies. It has been reported that a variety of ion currents can contribute to afterdepolarization in neurons. These ionic mechanisms include low threshold sodium currents, non-selective cationic currents, KCNQ/M channels, calcium-activated chloride currents, and sodium-calcium exchanger (Higashi et al., 1993; Linden et al., 1994; Azouz et al., 1996; Haj-Dahmane and Andrade, 1998; Yue and Yaari, 2004; Ghitani et al., 2016). As it would beyond the scope of the current study, future studies are needed to study the ionic mechanism of the novel afterdepolarization recorded in the present study.

One major finding of this study is that *in vitro* oxaliplatin selectively depolarized and increased the membrane excitability of the IB4^–^, but not IB4^+^ small DRG neurons. Previously, membrane depolarization and reduction in rheobases induced by *in vitro* oxaliplatin have been reported (Cerles et al., 2019; Zhang et al., 2021). However, a selective effect of *in vitro* oxaliplatin on the membrane excitability of IB4^–^ over IB4^+^ neurons has not been reported. As the two major subpopulations of DRG neurons, IB4^–^ and IB4^+^ neurons are largely different in their expression of neuronal peptides, peripheral innervation and central projection in spinal cord, modulation by growth factors, expression of ion channels and membrane receptors, and physiological function (Verge et al., 1989; Silverman and Kruger, 1990; Molliver et al., 1997; Zylka et al., 2005; Jankowski et al., 2009; Chiu et al., 2014). Therefore, a selective excitatory effect of oxaliplatin on the IB4^–^ subpopulation of small DRG neurons would selectively activate peptidergic innervation at periphery and therefore their central projections at Lamina I and outer Lamina II in the spinal cord.

In the present study, we found that a membrane-hyperpolarizing drug retigabine largely prevented the oxaliplatin-induced mechanical and cold hyperalgesia (Du et al., 2014). A previous study also showed that retigabine prevented oxaliplatin-induced orofacial cold hyperalgesia that is likely involved with TRP channels also (Abd-Elsayed et al., 2015; Luo et al., 2021). As retigabine opens potassium channels at resting membrane potential leading to membrane hyperpolarization, these results suggest that targeting oxaliplatin-induced membrane depolarization might be a useful strategy to prevent oxaliplatin-induced sensory hyperalgesia.

Another finding of the *in vitro* study is that blockade of TRPA1 and TRPM8, but not TRPV1 prevented the oxaliplatin-induced membrane depolarization and reduction in the rheobase of action potentials (Figure 4). These results suggest that TRPA1 and TRPM8, but not TRPV1 contribute to oxaliplatin-induced membrane depolarization in IB4^–^ subpopulation of small DRG neurons in mice. Previous it has been reported that *in vitro* oxaliplatin potentiates the increasing effects of agonists of TRPA1 and TRPV1, but not TRPM8 on intracellular Ca^2+^ in DRG neurons (Anand et al., 2010; Nassini et al., 2011; Zhao et al., 2012). The consistent (for TRPA1) and different (for TRPV1 and TRPM8) effects of TRP channel agonists on the membrane excitability compared to intracellular Ca^2+^ suggest that oxaliplatin might induce membrane hyperexcitability and increased intracellular Ca^2+^ through partially overlapping mechanisms in DRG neurons.

Upregulation of multiple TRP channel isoforms (TRPA1, TRPV1, and TRPM8) have been reported following oxaliplatin treatment (Chukyo et al., 2018). The upregulation of these TRP channels following oxaliplatin treatment may or may not contribute to the oxaliplatin-induced mechanical and/or cold hyperalgesia (Gauchan et al., 2009; Mizuno et al., 2014; Chen et al., 2015; Yamamoto et al., 2015). Different from previous studies, the current study is focused on the potential role of TRP channels and membrane depolarization as the initial ionic/membrane drives (such as within an hour) that contribute to the development of oxaliplatin-induced neuropathic pain. Our results suggest that TRPM8 and TRPM8-mediated membrane depolarization, but not TRPA1 or TRPV1, serve as the initial ionic/membrane drives that contribute to the development of oxaliplatin-induced neuropathic pain. On the other hand, although the current study might provide some new suggestion on the role of TRPM8 as the initial ionic drive for the development of oxaliplatin-induced acute pain, we fully recognize that the potential TRPM8 involvement in Oxaliplatin-induced neurotoxicity has been reported by other researchers previously (Gauchan et al., 2009; Ta et al., 2010; Descoeur et al., 2011; Kono et al., 2012; Mizuno et al., 2014; Hsieh et al., 2017; Chukyo et al., 2018).

Although most studies of TRPM8 and allodynia have been focused on the cold allodynia, the involvement of TRPM8 in mechanical allodynia has been reported in multiple inflammatory and neuropathic pain conditions (Gao et al., 2013; Caceres et al., 2017; De Caro et al., 2018). The current manuscript showed that TRPM8 also participant in the oxaliplatin induced mechanical allodynia in addition to the cold allodynia. However, a previous study found that knockout of TRPM8 did not prevent the development of mechanical hyperalgesia induced by oxaliplatin (Descoeur et al., 2011). This discrepancy might be due to the different mechanical force used for the von-Frey tests. The previous study used 1.4 g as the stimulating force while the current study found that the mechanical threshold changed from about 0.2 to 0.45 g in the absence and presence of TC-I pre-treatment. Moreover, it has been reported that a selective reduction of TRPM8, but not TRPV1 is associated with the transcutaneous ultrasound nerve stimulation-induced inhibition of mechanical and cold allodynia following oxaliplatin treatment (Hsieh et al., 2017). Therefore, it might be suggested that TRPM8 is more involved in the mechanical allodynia compared to mechanical hyperalgesia induced by oxaliplatin.

One inconsistence in results in the present study is that the TRPA1 antagonist prevented oxaliplatin-induced membrane depolarization in DRG neurons *in vitro* but did not prevent the oxaliplatin-induced neuropathic pain behaviors *in vivo*. One possibility for this discrepancy could be due to the dissociated cell culture of DRG neurons. It has been suggested that dissociation of DRG neurons might induce hyperexcitability that is caused by increased PKA and PKG signaling (Zheng et al., 2007). As PKA can sensitize TRPA1 channels, the TRPA1 *in vitro* might become more sensitive to oxaliplatin through ROS or other mechanisms compared to *in vivo* (Meents et al., 2017; Zheng et al., 2019). Another possibility might be that TRPA1 and TRPM8 are functionally coupled in the dissociated DRG neurons but not *in vivo*, as association of different types of TRP channels has been reported in DRG neurons (Patil et al., 2020). Nevertheless, other possibilities can not be excluded and future studies are needed to address this difference.

The present study suggests that oxaliplatin depolarizes IB4^–^ DRG neurons through activation of TRPM8 channels to drive the development of neuropathic pain in mice. However, a previous study suggested that oxaliplatin acts on IB4^+^ nociceptors to induce peripheral sensory neuropathy (Joseph et al., 2008). Specifically, the previous study found that intrathecal IB4-saporin prevented mechanical hyperalgesia induced by oxaliplatin while the present study found that the antagonist of TRPM8 (not expressed in IB4^+^ neurons) prevented both mechanical and cold hyperalgesia induced by oxaliplatin. Taken these results together, it is possible that oxaliplatin-induced mechanical hyperalgesia might be dependent on the activation of both IB4^+^ and IB4^–^ subpopulations of DRG neurons. On the other hand, in addition to the different blocking approach, there are multiple other conditions that might contribute to the different suggestions made from the previous and current studies. These differences include species of experimental animals (rat vs. mouse), route of administration of oxaliplatin (iv vs. ip), types of relevant behavioral tests (mechanical vs. mechanical and cold), and time points of relevant behavioral tests (6–10 vs. 3 days following oxaliplatin treatment).

## Data Availability Statement

The original contributions presented in the study are included in the article/supplementary material, further inquiries can be directed to the corresponding author/s.

## Ethics Statement

The animal study was reviewed and approved by the Institutional Animal Care and Use Committees of the Indiana University School of Medicine.

## Author Contributions

BW, XF, Y-HJ, and Z-YT conceived the idea. BW, XS, and Z-YT designed the experiments, analyzed the data, and wrote the manuscript. BW, XS, WZ, and Y-HZ conducted the experiments. All authors were involved in the discussion of the project.

## Conflict of Interest

The authors declare that the research was conducted in the absence of any commercial or financial relationships that could be construed as a potential conflict of interest.
